# Health care utilisation amongst Shenzhen migrant workers: does being insured make a difference?

**DOI:** 10.1186/1472-6963-9-214

**Published:** 2009-11-21

**Authors:** Jin Mou, Jinquan Cheng, Dan Zhang, Hanping Jiang, Liangqiang Lin, Sian M Griffiths

**Affiliations:** 1 Shenzhen Centre for Disease Control and Prevention, Shenzhen, 518020, Guangdong, China; 2 School of Public Health and Primary Care, The Chinese University of Hong Kong, Shatin, NT, Hong Kong; 3 Shenzhen Health Bureau, Shenzhen, Guangdong, China

## Abstract

**Background:**

As one of the most populous metropolitan areas in the Pearl River Delta of South China, Shenzhen attracts millions of migrant workers annually. The objectives of this study were to compare health needs, self-reported health and healthcare utilisation of insured and uninsured migrant workers in Shenzhen, China, where a new health insurance scheme targeting at migrant workers was initiated.

**Methods:**

A cross-sectional survey using multi-staged sampling was conducted to collect data from migrant factory workers. Statistical tests included logistic regression analysis were used.

**Results:**

Among 4634 subjects (96.54%) who responded to the survey, 55.11% were uninsured. Disease patterns were similar irrespective of insurance status. The uninsured were more likely to be female, single, younger and less educated unskilled labourers with a lower monthly income compared with the insured. Out of 1136 who reported illness in the previous two weeks, 62.15% did not visit a doctor. Of the 296 who were referred for inpatient care, 48.65% did not attend because of inability to pay. Amongst those who reported sickness, 548 were insured and 588 were uninsured.

Those that were insured, and had easier access to care were more likely to make doctor visits than those who were uninsured.

**Conclusion:**

Health care utilisation patterns differ between insured and uninsured workers and insurance status appears to be a significant factor. The health insurance system is inequitably distributed amongst migrant workers. Younger less educated women who are paid less are more likely to be uninsured and therefore to pay out of pocket for their care. For greater equity this group need to be included in the insurance schemes as they develop.

## Background

The relaxation of Hukou-residence-employment restriction in mainland China has contributed to increased population mobility and resulted in the largest rural-to-urban migrant population transition in the history of China [1,2]. Shenzhen, as one of the most populous metropolitan areas in the Pearl River Delta of South China, attracts millions of additional rural labourers annually. Many of these migrants are unskilled and minimally educated, migrating to the city in the hope of seeking employment and a better standard of living for themselves and their families [3]. They end up taking jobs far removed from their agricultural backgrounds. The local government estimated that by 2007, the overall population size of Shenzhen had reached 14 million people, of which around 60% were migrant labour workers from other regions of China. Migrant workers (MWs) generally have a lower income and poorer socio-demographic characteristics compared with the permanent residents of the city [4]. Major health disparities also exist between migrant workers and the local population as has been observed elsewhere [5-7]. The incomplete social security system and lack of health insurance makes the situation even worse [8]. Migrant workers are not entitled to Government Employee Insurance and Labour Insurance, which are the main types of health insurance for employees holding local *HuKou*, nor are they able to access the New Rural Cooperative Medical Insurance based on the fact that they live and work in the city.

Literature suggests that migration is associated with increased health risks related to occupational safety [9], infection [10,11], reproductive health [12], mental health [13-15] and health behaviours [10]. These vulnerabilities, together with low capacity to pay medical bills, poor access to healthcare, and resulting unsatisfactory health outcomes have been described by other researchers [5,16-18]. Former studies have also noted the potential societal and health consequences resulting from the ever-widening socioeconomic disparities [1,19].

Since migrants tend to be young and are presumed to have fewer chronic conditions when compared with their elders or peers left behind in the countryside [20], health-seeking behaviour plays an important decisive role in their health outcomes [21-24].

Early in 2004, the Shenzhen government issued a regulation concerning the development of a healthcare system to cater for migrant workers, recognizing that failure to provide adequate healthcare increases the risk of deepening health and social inequalities. From March 1^st ^2005, an experimental Cooperative Healthcare Service System for Migrant Workers (CHSMW) was initiated to provide coverage for services by contracting specific designated healthcare providers (DHP) in all 6 districts in our study [25]. In June 2006, CHSMW formally developed into the Medical Insurance System for Migrant Employees (MISM). This new system was open to all migrant workers in the city and is compulsory for employers.

According to the Ordinance of Labour Workers for the Shenzhen Special Economic Zone (SZSEZ), migrant workers who are eligible for the scheme are those employed by legal employers but who live in Shenzhen without permanent residential registrations (*HuKou*). The monthly contribution to CHSMW per individual worker is 12 RenMinBi (RMB), 8 RMB paid by employers and 4 RMB paid by the workers themselves. This personal contribution is quite low compared with their average monthly income (0.41% as of year 2005) [26]. Half of the fund (6 RMB) is designated for out-patient services and 5 RMB for in-patient services. A further 1 RMB is saved as an optional pool for further utilisation. The CHSMW scheme applies to designated healthcare providers (DHP) which include hospitals and community centres that are designated through a formal accreditation process undertaken by the municipal or district Health Bureau and licensed by the city's Social Security Bureau (SSB). Payment to each DHP is based on the number of migrant workers for whom the DHP provides outpatient services. Inpatient service reimbursement and referral is managed by the SSB through standardized procedures. Since June 2006, MISM has become compulsory in all 6 districts of the city. The new scheme changed the collection requirements to 0.45% of the previous year's average monthly salary per capita for employed workers. Employers contribute 0.3% and individual workers contribute 0.15% into the fund. Both CHSMW and MISM ensure 60% to 80% reimbursement for category I and category II medicines, treatments, examinations and consultations in outpatient services while covering 60% to 100% reimbursement of inpatient services utilised depending on the price per item and level of DHP concerned. A very minimum registration fee is charged each time the insured workers access DHP. By the end of 2006, the MISM reported coverage of 3 million migrant workers in Shenzhen and has a significantly higher percentage of insured migrant workers compared with the CHSMW, although both schemes have been subsidised by the city government. Although all employers are now required to pay health insurance fees for employed migrant workers, the scheme has failed to include all enterprises due to difficulties in enforcement. The decision to join the MISM scheme is largely in the hands of the employers and not the employees, which itself raises questions about equity and the each individual's right to health and health care services.

In this paper, we present the results of a cross-sectional survey conducted on a representative sample of migrant workers in Shenzhen, China between March 2005 and May 2005 during the trial period of the CHSMW scheme. The main objectives were to describe and compare socio-demographic characteristics, self-rated health (SRH) and health service utilisation patterns amongst both insured and uninsured migrant workers.

## Methods

### Sampling

A multi-staged random sampling approach to obtain the study population was adopted. Four sub-district areas were randomly selected and 30 legally registered enterprises were then randomly selected from each selected sub-district. Features of enterprises sampled varied substantially from garment and shoes making to optical instruments and electronic hardware manufacturing. Employee varied between 90 in some small factories to more than 7000 in those big international corporations. 40 migrant workers between 18-60 years old were randomly selected for interview from each selected factory or company, resulting in 4800 MWs in the study. This sample was compared with data from the 2005 Census on Migrant Worker released by Shenzhen Health Bureau and no statistically significant differences were found between for sex, age, and education.

### Data collection

All eligible subjects were asked to complete a standardized questionnaire administered by licensed physicians who were trained under a standard interview procedure. The questionnaire was derived from an original questionnaire designed for use amongst general Chinese populations by the Ministry of Health of China [27]. Information obtained included each worker's socio-demographic characteristics (age, sex, education, marital status, occupation, monthly income, living expenses, medical expenditure, and current health insurance status of the recent year), self-rated health, outpatient and inpatient healthcare utilisation, and features of the nearest/most frequently used health facilities. A letter explaining purpose of the survey and an informed consent form were distributed to the selected MWs before the questionnaire was given. It was required that the participants read and sign the consent form. The study was approved by the Institutional Review Board of Shenzhen Health Bureau (No. 200501028).

### Measurement

A subjectively dichotomized scale was used to measure the respondent's SRH. Episodes of illness in the preceding past two weeks and the prevalence of long-term illness/disorders in the past six months were recorded. Self-perceived reasons for not pursuing healthcare and/or refusal to hospital admission were also collected. For those who had ever utilised healthcare facilities during the study time period, the features of the health facilities were asked. To include all possibilities, we provided multiple-choice options and open questions. Data on self-reported diseases were further confirmed by crosschecking the medical records kept by the interviewees, reimbursement records, or by communication and were then coded into categories in accordance with the 10^th ^revision of the International Classification of Diseases (World Health Organization, 2008) [28] by trained doctors.

### Data analysis

Chi-square tests were used to compare the different proportions of categorical variables. To control for the potential confounding effects of socio-demographic factors, multiple logistic regression models were used to estimated the adjusted odds ratio and the 95% confidence intervals of having visited doctors in the previous two weeks with socio-economic status, SRH, sickness of previous two weeks, presence of long-term illness, physical accessibility to community health centres, characteristics of the nearest health facilities, and health insurance participation. Data were analyzed using SPSS 16.0 (SPSS Inc, Chicago, Illinois, USA).

## Results

Among 4800 workers selected 4634 (96.54%) completed the questionnaire. More than half of the participants were female (59.26%), aged 18-24 years (51.27%), received junior high school education (56.63%), married (59.82%), general unskilled labourers (52.68%), and had a monthly income of the category 500-999 RMB (51.75%). Table 1 shows the socio-demographic characteristics by health insurance status. Only 44.89% participants were reported having participated in the insurance scheme during the past 12 months. The uninsured were likely to be female, younger, less educated, single, general unskilled labourers, and having a lower monthly income.

**Table 1:** Socio-demographic characteristics of migrant workers by insurance status, Shenzhen, China

Demographic Characteristics	Insured (n = 2080)		Uninsured (n = 2554)		Total (n = 4634)	
	n	%	n	%	n	%
Sex						
Male	883	42.45	1005	39.35	1888	40.74
Female	1197	57.55	1549	60.65	2746	59.26
*p *= 0.033 *χ*^2 ^= 4.569						
Age						
18--24	889	42.74	1487	58.22	2376	51.27
25--34	872	41.92	784	30.70	1656	35.74
35+	319	15.34	283	11.08	598	12.99
*p *= 0.000 *χ*^2 ^= 110.003						
						
Education						
Illiterate	8	0.38	25	0.98	33	0.71
Primary	66	3.17	94	3.68	160	3.43
Junior high school	1056	50.77	1568	61.39	2624	56.63
Senior high school	705	33.89	722	28.27	1427	30.79
College	192	9.23	116	4.54	276	6.65
University or above	53	2.55	29	1.14	82	1.77
*p *= 0.000 *χ*^2 ^= 92.019						
						
Marital status						
Single	708	34.03	1349	52.81	1847	39.86
Married	1364	65.60	1081	42.33	2772	59.82
Others	19	0.91	124	4.86	15	0.32
*p *= 0.000 *χ*^2 ^= 266.095						
Occupation						
Administrative	341	16.39	327	12.80	668	14.42
Clerk/technician	591	28.41	638	24.98	1229	26.52
General worker	1051	50.53	1390	54.42	2441	52.68
Others	97	4.66	199	7.79	296	6.39
*p *= 0.000 *χ*^2 ^= 36.214						
Monthly Income (CNY)						
< 500	26	1.25	108	4.23	134	2.89
500--999	965	46.39	1433	56.11	2398	51.75
1000-1499	684	32.88	611	23.92	1295	27.95
1500-1999	206	9.90	202	7.91	408	8.80
2000+	199	9.57	200	7.83	399	8.61
*p *= 0.000 *χ*^2 ^= 98.215						

Amongst 4634 participants, 24.51% (1136 persons, 548 were insured and 588 were uninsured, 95%CI: 24.45%-28.23%) had reported diseases or discomforts in the preceding two weeks, slightly higher (*χ*^2 ^= 6.843, *p *= 0.009) for the insured (26.34%, 95%CI: 24.63%-28.05%) than the uninsured (23.02%, 95%CI: 21.39%-24.65%), although subgroups of the uninsured with minimum education showed comparatively high proportion of reporting. Amongst those reporting any illness during the previous 2 weeks, the distribution of some demographic factors was significantly different within the insured and uninsured groups (Table 2). Younger general workers were less likely to report illness in the previous two weeks in both groups.

**Table 2:** Migrant workers' self-reported two-week-illness by insurance status and socio-demographic factors

Factors	Insured (548 reports, incidence rate = 26.34%)				Uninsured (588 reports, incidence rate = 23.02%)			
	*n*	%	*χ*^2^	*p*	*n*	%	*χ*^2^	*p*
*Sex*			0.93	0.3345			3.36	0.0700
Male	242	27.40			253	25.17		
Female	306	25.56			335	21.63		

*Age*			14.49	0.0007			14.01	0.0009
18-24	197	22.16			308	20.38		
25-34	259	29.70			184	22.83		
35 or above	92	28.84			96	30.39		

*Education*			23.22	0.0003			25.31	0.0001
Illiterate	1	12.50			12	48.00		
Primary	8	12.12			28	29.79		
Junior high	238	22.53			309	19.71		
Senior high	227	32.20			197	27.29		
College	47	24.48			30	25.86		
University	7	13.21			12	41.38		

*Occupation*			33.04	<0.0001			38.07	<0.0001
Administrative	119	34.90			100	30.58		
Clerk/technician	167	28.26			176	27.59		
General worker	220	20.93			266	19.13		
Others	22	22.68			46	23.12		

The top five causes of self-reported diseases were acute upper respiratory infections (J06.9, 760 persons, 16.40%), acute and chronic gastritis (K29, 150, 3.24%), acute nasopharyngitis (J00, 59, 1.27%), inflammatory diseases of female pelvic organs or non-inflammatory disorders of female genital tract (N70-N77, 57, 2.07%), and injuries (S00-T32, 35, 0.76%), accounting for 93.39% of the overall reports totally and acute upper respiratory infections were the most likely reasons (66.93%). No significant differences in the types of medical complaint were observed between the insured and uninsured.

We defined long-term illness as any disease that had lasted for more than 2 weeks in the past 6 months. The overall prevalence of self-reported long-term illness diagnosed by doctors was 18.64% (95%CI: 17.52%-19.76%, 864 subjects). No significant difference was observed between the insured and uninsured. The Top ten causes included: nonorganic insomnia (F51.0, 206 reports, 4.45%), disorders of refraction and accommodation (H52, 174, 3.75%), gastric ulcer or duodenal ulcer or chronic gastritis (K25, K26 & K29.5, 164, 3.54%), migraine (G43, 164, 3.54%), other intervertebral disc disorders (M51, 105, 2.27%), depressive episode and unspecified anxiety disorders (F32 & F41.9, 91, 1.96%), chronic bronchitis (J41-42, 79, 1.70%), gonarthrosis or arthrosis of the knee (M17-M19, 61, 1.32%), dental cavities (K02, 41, 0.88%), and other respiratory diseases (J95-J99, 40, 0.86%).

Amongst those reporting any illness in the previous two weeks (*n *= 1136), 430 (37.85%) had visited a doctor whilst 462 (40.67%) had used self-treatment. 235 (20.69%) did not take any measure and 9 (0.79%) reported other management methods for illnesses. In total, 851 medical consultations were reported by 430 workers ever visiting doctors in the past two weeks, reflecting a two-week doctor visit rate of 18.36% (851/4634). The insured sick workers had a significantly higher rate of doctor visit (242/548 vs. 188/588, *χ*^2 ^= 16.99, p < 0.0001) but less frequent per person consultations than the uninsured workers (242 persons had 443 consultations, 1.83 per user, 188 persons had 408 consultations, 2.17 per user, *p *< 0.05) in the past 2 weeks.

Amongst all the MWs who were seeking health care, the majority presented to community health centres (34.88%), private clinics (20.93%) and government-run sub-district level hospitals (20.00%). Differences in utilisation rates by insured and uninsured across the range of healthcare institutions, excluding private hospitals which mainly deal with plastic surgery, were all statistically significant. The insured tended to use community health centres more frequently, while the uninsured tended to use private clinics the most and pay out of pocket (Table 3). In total, out of the 39 different private clinics used by 61 uninsured workers, 34 (87.18%) had no license issued by any health authority and use of intravenous antibiotics was the main and only treatment (48/61, 78.69%).

**Table 3:** Characteristics of health facilities used for outpatient services by MWs by health insurance status

Characteristics of health facility used	Insured (n_1 _= 242)		Uninsured (n_2 _= 188)	
	n	%	n	%
Health insurance designated community health centres *p *< 0.01	106	43.80	44	23.40
Private clinics *p *< 0.01	29	11.98	61	32.45
Sub-district level hospitals *p *< 0.05	54	22.31	32	17.02
City level medical centres *p *< 0.01	12	4.96	23	2.23
District hospitals *p *< 0.05	14	5.79	15	7.98
Employer-run clinics at worksite *p *< 0.01	16	6.61	3	1.60
Private hospitals	6	2.48	4	2.13
Provincial hospitals or medical centres *p *< 0.05	3	1.24	1	0.53
Others	2	0.83	5	2.66

Total	242	100.00	188	100.00

Of the 1136 workers who reported illness, 706 (62.15%) chose not to visit a doctor. Of those not seeking a doctor, 65.44% chose self-treatment while 33.29% took no measure. The main reasons influencing migrant workers' adoption of self-treatment included (multiple answers allowed): believing the local pharmacy was very convenient (53.10%) and perception of illness not being severe (51.60%). Lack of spare time (25.31%) and being unable to afford care (25.61%) were also cited as major reasons. A smaller proportion (8.98%) did not seek medical attention citing poor service provision at health facilities as their main reason. Only 20 believed that lack of effective treatment made them decline doctor-visit (8.16%).

In total, 152 hospital admissions were recorded in the previous year (3.28%), mainly at sub-district level (39.84%), city-level (27.64%) and district-level hospitals (21.14%). Nearly half (48.65%) of the patients for whom hospitalisation was recommended did not get admitted. The main reasons given included not being able to pay, a reason not only applicable to the uninsured since many insured were unable to pay up front prior to reimbursement by the insurance system (68.06%). Other reasons given included lack of time (33.33%), self-perception of needlessness (29.86%) and poor previous experience in getting health services (11.81%).

Table 4 compares the associations of socio-demographic factors with doctor visit in the past 2 weeks by using multiple logistic regressions. We found that increased doctor visit was significantly associated with health facilities offered by employers, physical accessibility to community health centres affiliated to health insurance, being insured and better self-rated health.

**Table 4:** Logistic regression model for factors influencing doctor-visit in previous two weeks

				Adjusted OR		
				
variables	*b*	*χ*^2^	*p*	Estimates	95% Confidence Intervals	
Constant	-1.3346	24.6943	0.0001	------	------	

Senior high school education	-0.4219	8.9117	0.0028	0.656	0.497	0.865

Health facilities offered by employers	0.9464	7.0661	0.0079	2.576	1.282	5.177

The nearest facilities being community health centres	0.0709	7.9383	0.0048	1.588	1.214	2.075

Being insured	0.3674	6.6807	0.0097	1.444	1.093	1.908

SRH	0.2188	6.2283	0.0126	1.244	1.048	1.476

## Discussion

The New Rural Cooperative Medical System (NRCMS) was introduced in 2003 and aimed to cover migrants whose original *HuKou *are in rural locations. However rural migrants living and working in cities actually can not benefit from NRCMS. In Shenzhen the CHSMW was introduced as a pilot to redress this problem. One major feature of Shenzhen's pilot was to include more community health centres as DHP so as to encourage migrant workers' utilisation of health at community level. This study was undertaken to help shape the development of health insurance in Shenzhen. Our study has shown that a high proportion of MWs in Shenzhen were uninsured, and that insurance status was lower amongst the socio economically disadvantaged. It would appear that employers were more likely to pay insurance contributions for more advantaged workers (more experience, stable, male and better educated) while not contributing to health insurance for those with disadvantage (less educated, female, new thus more mobile). Some employers established criterion to select "qualified" workers for the scheme. MWs rates of two-week illness and long-term illness were higher than those found in national surveys [27]. These were unexpected findings in the context of the traditional paradigm which perceives younger individuals as healthier and less likely to develop illness.

The commonest self reported long-term health problems in this study could be explained by the socioeconomic environment in which migrant workers are living including working long hours under pressure, poor working and living conditions or being trapped in an ambiguous grey social zone. Services offering consultations for psychological problems, assessment and improvement of working environments, and alleviation of working pressures are not readily available. Earlier studies have found factors affecting service utilisation ranged from cultural and socio-demographic factors, physical accessibility, disease patterns to perception of quality of services and confidence in care [29]. In our study the insured and uninsured differed in healthcare utilisation. The insured were more likely to pay doctor visits when sick and use health care in community health centres but had less visits per episode compared with the uninsured who tended to seek care less frequently when sick, go to private clinics, pay out-of-pocket and have more visits per episode once they chose to use services. The possible implication for the uninsured to choose private clinics, most of which are unlicensed, is the physical convenience and being unable to access the better quality and more appropriate services available to the insured. Another contributing factor might be that the uninsured had lower levels of awareness of other options compared with their insured peers who were given a list of all DHPs that they were entitled to access. Once the treatment in private clinics is initiated, MWs face higher costs because they have more frequent consultations per episode and are more likely to be referred to higher-level hospitals where they face paying expensive bills in. Other factors which may influence the utilisation pattern include the generally lower quality of private doctors' practice or unsatisfactory outcomes of treatments. Since the scheme was semi-compulsory and payment of premium was very small, adverse selection seemed to be quite low. However, we were not clear about the existence of moral hazard affecting health utilisation of the insured.

Migrant workers were more likely to see a doctor when facilities are offered by employers, when the nearest care facilities were local community health centres and when they were insured. Better access to community health care would appear to encourage more appropriate use of health services as found elsewhere [30]Accessible and appropriate care are key for developing the primary health care system and improving coverage of health insurance among MWs. Another factor which needs to be addressed is financial affordability of healthcare which is not yet satisfactory for MWs despite their insurance status. This is especially obvious in inpatient care use. A statistic report released in 2006 showed the average inpatient care cost per person-time was 4745.5 RMB [31], whilst the average monthly income in that year was only 970 RMB. Aside from remittance they sent home and living expenses, migrant workers often find it hard to raise enough money for the hospital deposit even if they are insured. In another audit study MWs reported "being unable to pay" as the major reason of not using both outpatient and inpatient care [32]. This finding was consistent with our study and implies that introduction of the CHSMW has not eliminated the financial barrier to care for the participants, although to some extent it has improved health utilisation amongst the insured. Adjustment of reimbursement mechanisms and the increase of government input may be important, though some other factors may also be considered

The importance of physical accessibility [33] was further underlined by our findings. Due to strict management directives of employers and routine overtime working schedules, it is often difficult for MWs to obtain leave to see a doctor far from their worksites. Affiliated community health centres are more convenient and closer than comprehensive hospitals and thus provide a better fit for their basic care needs. Physical distance to and opening hours of new community health centres are key considerations within the strategy for comprehensive health services.

Utilisation patterns within the insured workers group remain inconsistent. This may be due to the complex interaction of factors amongst which insurance status is only one factor and is worth exploring further. Rates of hospitalisation rejection as well as perceived inability to pay were still high. Contributing factors could be an unsatisfactory reimbursement system, poor knowledge on how to use health care properly, lack of time, and poor judgment of illness. Further discussion between workers, employers, health insurance providers and affiliated health centres is required.

One of the limitations of our study is that we used a cross-sectional design, this precluded adjusting for the different frequencies of disease/discomfort reports and types of disease episodes, both of which varied according to seasons and relative work intensity. This also means that we could not track variations of insurance status of the migrants in the cases of frequent job change or high mobility. In fact, in the Pearl River Delta area, a portion of migrant workers do change jobs on a yearly or even seasonal basis. Perhaps in the future a longitudinal study design to track migrants and their health-related behaviours may be more effective through following-ups.

The risk of misclassifying data in terms of disease status can be noted as another potential limitation. This bias may be the result of episode reports collected on the basis of self-reporting and some minimal physical measurements taken by the interviewers. Some chronic conditions, if in the early stage of development, may have gone unnoticed since laboratory tests were not utilized. There is thus the potential for under-estimation of the prevalence rates of some chronic diseases, for example cancers.

The third limitation that needs to be addressed by future studies is that the inclusion of only those migrants with occupations in productive industries, the dominant industry in the city of Shenzhen. This sampling choice has therefore excluded all workers in other fields like the tertiary or service industry and construction. It is also worth noting that we have not investigated if there are any links between self-reporting (subjective measure) and the reimbursement data (objective measure) in the Health Security Bureau for those that are insured. The reason for not doing this is the technical barrier that complicates linking anonymous data.

Finally, since MISM in Shenzhen is still evolving. Some new features have been added and enforcement actions have been taken since 2006, in order to promote participation of more disadvantaged workers, particularly new and female workers.

## Conclusion

Providing appropriate healthcare services for MWs in China is one of the priorities in terms of economic development and social stability. Despite attempts to improve access to healthcare by providing health insurance for migrant workers our study has shown that insurance is not universally made available by employers who selectively provide insurance to the more affluent amongst the working community. Younger less skilled women are often without insurance. Their expenditure on health care is higher per episode and the costs associated with attending private unregistered clinics leads to a higher financial burden. This impacts both on themselves and their families in rural areas who are often dependent on their incomes. Modifying the insurance scheme to ensure equitable access to care in community is important as is reconsideration of the methods for reimbursement. Our study found that the health insurance system is inequitably distributed amongst migrant workers, resulting in disparities in health utilisation. Inability to pay and high uninsured rates suggest the need to reform and continue to evaluate the developing health insurance systems for migrant workers in the city.

## Competing interests

We declare that we have no financial, personal relationship or any other non-financial competing interests with other people or organizations that can inappropriately influence our work. There is no professional or other personal interest of any nature or kind in any product, service and/or company that could be construed as influencing the position presented in, or the review of, the manuscript. We confirm that there are no known conflicts of interest associated with this publication and there has been no significant financial support for this work that could have influenced its outcome.

## Authors' contributions

JM helped with literature review, statistics and manuscript preparation. JC, HJ, DZ designed and participated in the field work of the study. Liangqiang Lin, helped with data collection and statistic analysis. SMG gave the research careful advice and revised the manuscript. All authors read and approved the final manuscript.

## Pre-publication history

The pre-publication history for this paper can be accessed here:

http://www.biomedcentral.com/1472-6963/9/214/prepub
